# sEMG Sensor Using Polypyrrole-Coated Nonwoven Fabric Sheet for Practical Control of Prosthetic Hand

**DOI:** 10.3389/fnins.2017.00033

**Published:** 2017-02-06

**Authors:** Yinlai Jiang, Masami Togane, Baoliang Lu, Hiroshi Yokoi

**Affiliations:** ^1^Brain Science Inspired Life Support Research Center, University of Electro-CommunicationsTokyo, Japan; ^2^Department of Mechanical Engineering and Intelligent Systems, University of Electro-CommunicationsTokyo, Japan; ^3^Department of Computer Science and Engineering, Shanghai Jiao Tong UniversityShanghai, China

**Keywords:** surface electromyography, myoelectric control, prosthetic hand, conductive polymer, electrode

## Abstract

One of the greatest challenges of using a myoelectric prosthetic hand in daily life is to conveniently measure stable myoelectric signals. This study proposes a novel surface electromyography (sEMG) sensor using polypyrrole-coated nonwoven fabric sheet as electrodes (PPy electrodes) to allow people with disabilities to control prosthetic limbs. The PPy electrodes are sewn on an elastic band to guarantee close contact with the skin and thus reduce the contact electrical impedance between the electrodes and the skin. The sensor is highly customizable to fit the size and the shape of the stump so that people with disabilities can attach the sensor by themselves. The performance of the proposed sensor was investigated experimentally by comparing measurements of Ag/AgCl electrodes with electrolytic gel and the sEMG from the same muscle fibers. The high correlation coefficient (0.87) between the two types of sensors suggests the effectiveness of the proposed sensor. Another experiment of sEMG pattern recognition to control myoelectric prosthetic hands showed that the PPy electrodes are as effective as Ag/AgCl electrodes for measuring sEMG signals for practical myoelectric control. We also investigated the relation between the myoelectric signals' signal-to-noise ratio and the source impedances by simultaneously measuring the source impedances and the myoelectric signals with a switching circuit. The results showed that differences in both the norm and the phase of the source impedance greatly affect the common mode noise in the signal.

## Introduction

Robotic prosthetic hands have benefitted from significant improvements due to the quick advance of robotics and biomedical engineering in the last decade. The prosthetic hand is expected to restore the lost hand's function through hardware that can operate like a human hand and an interface that can transfer the user's motion intention to the hardware. In regard to the hardware, many dexterous prosthetic hands with multiple degrees of freedom (DoFs), such as the Vincent hand by Vincent Systems, the iLimb hand by Touch Bionics, the Bebionic hand by RSL Steeper, and the Michelangelo hand by Otto Bock, have been developed and are commercially available (Belter et al., 2013). In regard to the human interface, electromyography (EMG) signals associated with residual muscle contraction, from which the user's motion volition is recognized according to the remnant muscle activity, have been studied (Asghari Oskoei and Hu, 2007). Studies on the classification of EMG signals have reported remarkable results with advanced algorithms based on machine learning methods such as nonnegative matrix factorization, linear regression, linear discriminant analysis, support vector machines, and artificial neural networks (ANN), which have been proposed to identify as many patterns as possible from limited EMG sources and to deal with the unsteadiness, nonlinearity, individual difference, and low reproducibility of EMG signals (Al-Timemy et al., 2013; Young et al., 2013; Amsüss et al., 2014; Hahne et al., 2014; Jiang et al., 2014b; Earley et al., 2016).

However, despite the significant improvement of the mechanics and the EMG analysis methods, most of the currently commercially available myoelectric prosthetic hands used by amputees are still controlled by very simple algorithms that process the surface electromyogram (sEMG) to control 1°C of freedom (DoF) at a time. Threshold-level control, which generates a command to control one motion with one EMG source according the signal amplitude, is still the most widely used method (Dosen et al., 2010; Young et al., 2012). The main problem for applying advanced algorithms to control dexterous prosthetic hands in real life is the difficulty of measuring stable myoelectric signals. There are many factors, such as electrode shift (Young et al., 2012; Pan et al., 2015), noises in the signals (Chowdhury et al., 2013), and fatigue that causes the change of EMG (Beck et al., 2014), for the fault of applying advanced algorithms. Some studies turn to multi-modal sensing to overcome the difficulties of myoelectric control (Fang et al., 2015). The difficulty to measure useful signals with a high signal-to-noise (S/N) ratio using the current measurement methods is one of the main reasons for keeping amputees away from the advanced prosthetic hands. Most of the algorithms reported are tested in labs with only high-quality signals which are unavailable for used by people in their daily lives. Furthermore, the shape, size, and site of the residual limbs are different from one amputee to another, especially for young amputees whose residual limbs are still changing with their growth (Vasluian et al., 2013). Commercially available sEMG sensors allow little customization to adapt to these various and variable conditions. In real-life applications, the differences caused by analysis algorithms are less than those caused by measurement methods, since the quality of the EMG signal determines the usability and the stability of the myoelectric control.

A sEMG sensor is an electrochemical transducer that detects biopotentials by using electrodes placed on the skin. Since the measured potentials have small amplitudes, <30 mV when measured on the muscle fiber and <1 mV when measured on the skin surface, the potentials are susceptible to electromagnetic interference. Consequently, circuits that amplify the signals and filter external noise are usually required. A traditional way to measure EMGs for myoelectric control is differential amplification, in which the electrodes and the amplifier are the pre-amplifier and are the most important factors affecting the S/N ratio. Ag/AgCl electrodes, so-called “wet electrodes” utilizing an electrolytic gel to form a conductive path between skin and electrode, are currently the most widely used in hospitals and laboratories. Despite the fact that the low electrode-skin impedance of Ag/AgCl electrodes contributes to a high S/N ratio, dry electrodes made from metals, e.g., MyoBock 13E200-50 electrodes (Otto Bock HealthCare GmbH) made of pure titanium, are always adopted in real-life myoelectric control, since wet Ag/AgCl electrodes are disposable and not suited for long-term measurement. Electrodes reported recently include implantable electrodes (Merrill et al., 2011), invasive fine wire electrodes (Smith et al., 2016), low invasive percutaneous implant (Hahne et al., 2016), noninvasive metal electrodes with special curved microneedle array-based structures (Kim et al., 2015), and high-density multichannel electrodes (Popovic-Maneski et al., 2013). The implantable and invasive electrodes are suitable for measuring high-quality signals for dexterous control and diagnosis in hospitals, but the risk of infection makes them difficult for long-term everyday use. Most of the noninvasive electrodes made of metals have a fixed shape and size and are not customizable.

The objective of this study is therefore to develop usable and customizable sEMG sensors for practical control of a prosthetic hand in daily life. In preliminary studies, we developed both multi-DoF prosthetic hands with an interactive-wire driven mechanism (Seki et al., 2013; Morishita et al., 2014) and two DoF prosthetic hands simplified for quick practical use (Jiang et al., 2014a). An fMRI study with amputees showed the adaptation process in the brain for control of the prosthetic hand (Kato et al., 2009). However, for daily use of the prosthetic hand by a person with a disability, the number of recognizable sEMG patterns is limited by the usability and stability of the sEMG electrodes. This study introduces a sEMG electrode made of polypyrrole-coated nonwoven fabric sheet (PPy electrode) that can be easily customized to any person and can be worn one-handed for stable measurement of sEMG signals. Preliminary results on the PPy electrodes, including the manufacturing method, and basic performance for the control of myoelectric prosthetic hands, were reported in conference proceedings (Jiang et al., 2015). This study further tested the PPy electrodes by amputees and compared them with traditional disposable wet Ag/AgCl electrodes. Furthermore, we investigated the relation between the S/N ratio and the source impedances when using the PPy electrodes by simultaneous measurement of the source impedances and sEMG signals. The results showed that source impedances can serve as a useful reference when manufacturing the PPy electrodes. The possibility of improving the S/N ratio by balancing the source impedances was also indicated.

## Materials and methods

### Development of the sEMG sensor

#### Electrode material

**Figure 1:**
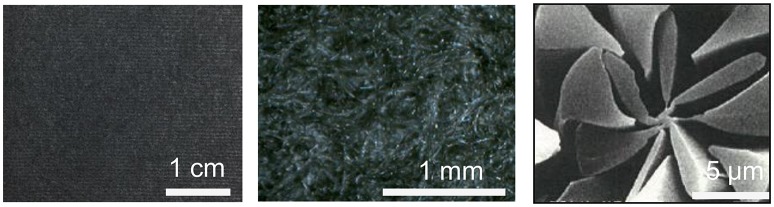
**Polypyrrole-coated nonwoven fabric sheet**.

A sEMG sensor using polypyrrole-coated nonwoven fabric sheet (Japan Vilene Company, Ltd) as electrodes (hereinafter referred to as PPy electrodes) is proposed for people with disabilities to control prosthetic limbs in daily life. The material is shown in Figure 1. The base material is polyethylene terephthalate and nylon, coated with polypyrrole, a kind of conducting polymer. The thickness of the sheet is 0.5 mm and the surface resistivity is <1 × 10^5^ Ω/sq. Compared with poly(3,4-ethylenedioxythiophene)-poly(styrenesulfonate) (PEDOT-PSS), which has been reported for electrodes measuring bioelectrical signals (Yamato et al., 1995; Richardson-Burns et al., 2007; Lang et al., 2009; Tsukada et al., 2012; Isik et al., 2015; Nagamine et al., 2016; Pani et al., 2016; Papaiordanidou et al., 2016), the conductivity of polypyrrole is lower, but it is more stable in an ambient environment that may be humid and wet due to skin sweating with a lower water-solubility and a lower hygroscopicity (Lee et al., 2001; Jang et al., 2004; Xu et al., 2006; Lang et al., 2009; Elschner et al., 2010).

#### Structural design of the sensor band

**Figure 2:**
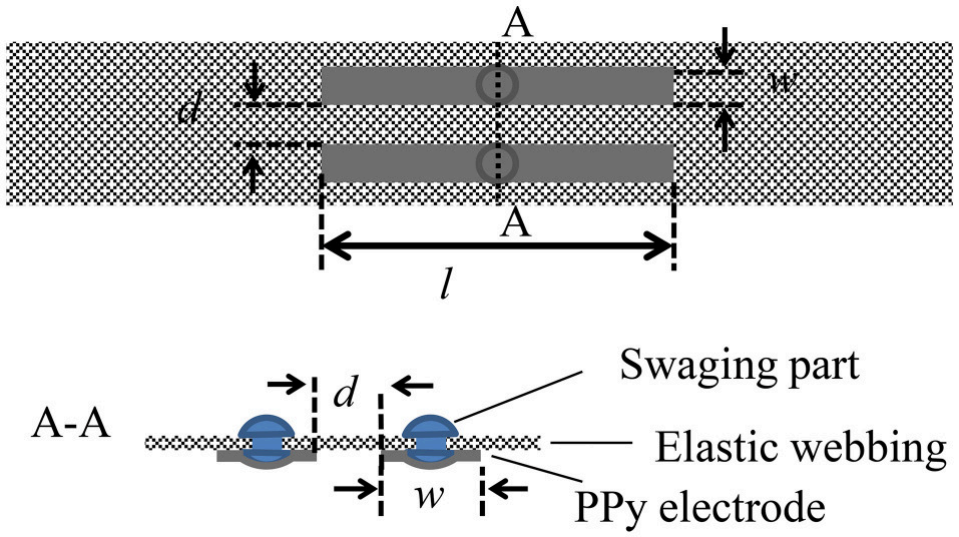
**Structural design of PPy electrode**.

PPy electrodes are easily customized to fit the size and the shape of the residual parts of an amputee. To attach PPy electrodes to the stump and to keep them on the skin, the PPy electrodes are sewn on an elastic band, as shown in Figure 2. Length l and width *w* of the electrodes, and distance *d* between pairs of electrodes can be adjusted to match a specific user. The elasticity of the band leads to close contact to the skin, and thus reduces the skin-electrode impedance and enables stable sEMG measurement with a high signal-to-noise ratio. Stainless swaging parts are used to transduce the electrical potentials from the PPy electrodes to the circuit of amplifier and filter.

### Trial use

Myoelectric control of a prosthetic hand with PPy electrodes was tested by a 14-year-old girl with a congenital right upper limb deficiency and a 36-year-old man with a right upper limb amputation that happened 5 years earlier. All the experimental protocols of this study were approved by the ethics board of the University of Electro-Communications [No. 10006(4)]. All subjects were fully informed about the procedures, risk, and benefits of the study, and written informed consent was obtained from all subjects before the study.

**Figure 3:**
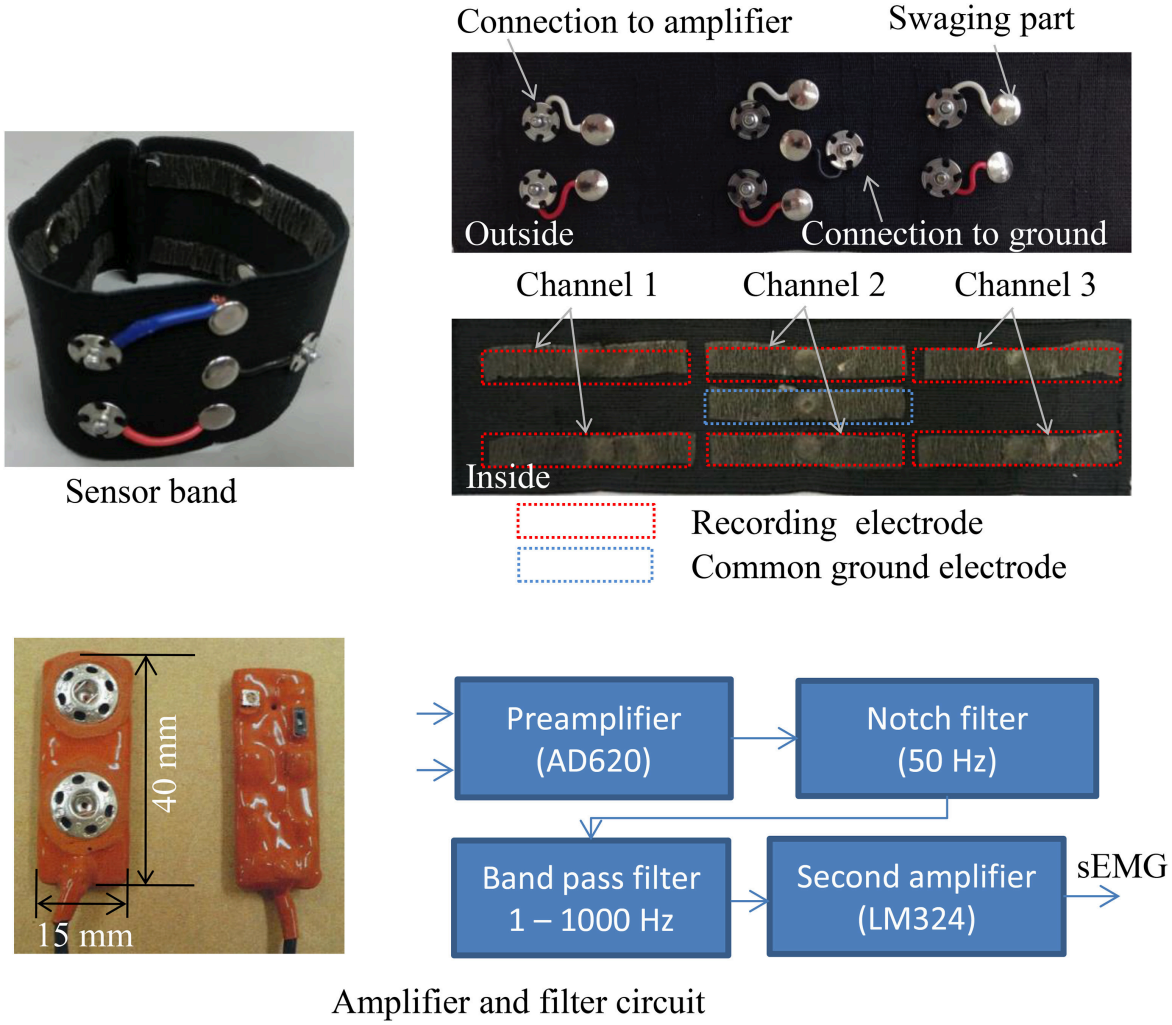
**Sensor band composed of PPy electrodes**.

Figure 3 shows the 3-channel sEMG sensor band with six recording electrodes for differential amplification and a common ground electrode. The potentials detected by the PPy electrodes were amplified and filtered by the circuit shown in Figure 3. The circuit was connected to the electrodes as closely as possible to ensure the electrical contact avoided noise. The circuit consisted of a preamplifier using an instrument amplifier AD620 (Analog Devices, USA) with a gain of 40 dB, a band pass filter with a 1–1000 Hz bandwidth, a 50 Hz notch filter, and a second amplifier. The output voltage range of the circuit was (−9 v, 9 v).

**Figure 4:**
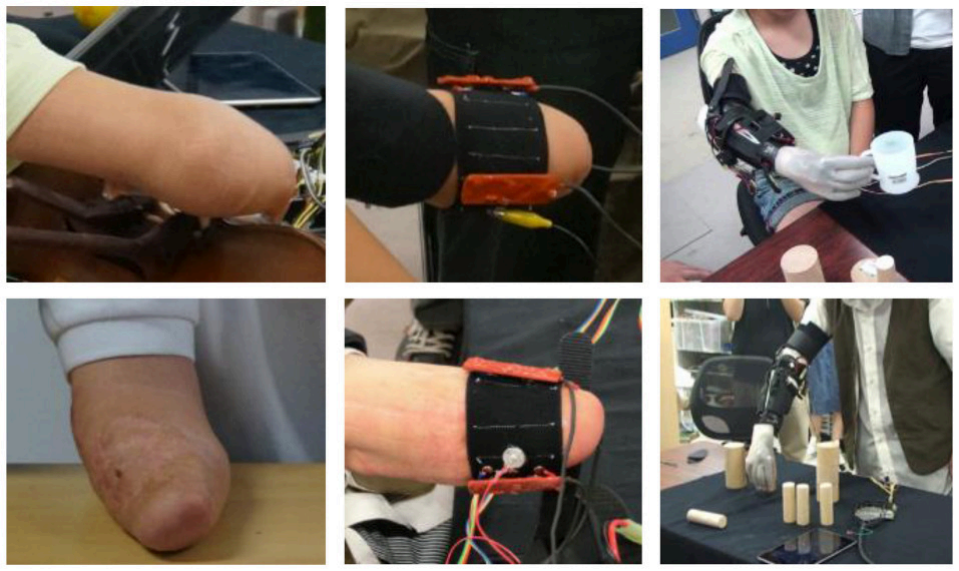
**Controlling prosthetic hand with the proposed PPy electrodes**.

The subjects wearing the sensor band tried to control a prosthetic hand developed in a preliminary study (Jiang et al., 2014a). The prosthetic hand consisted of a lightweight robotic hand with two motors to realize power grasp, precision grasp, and lateral grasp, a socket to mount the hand to the residual forearm, and a controller (SH72544R, Renesas Electronics Corporation, Japan) that performed EMG signal processing, pattern recognition, and motor control. The sEMG sensor band was worn on the stump covered by the socket. Figure 4 shows that the stumps of the subjects are quite different. They were able to one-handed attach the sensor bands customized for them by themselves. With the sensor bands, they tried to control the myoelectric prosthetic hand to perform some daily task, e.g., picking up a cup or building blocks, as shown in the figure.

## Comparison with wet Ag/AgCl electrodes

The trial by the subjects illustrated the practicability of the sensor band. To further verify the performance and characteristics of the PPy electrodes, these electrodes were compared with traditional wet Ag/AgCl electrodes in two experiments: one to investigate the correlation of the measured sEMG signals and the other to compare the accuracy of sEMG signal pattern recognition for myoelectric control.

### Analysis of signal correlation

**Figure 5:**
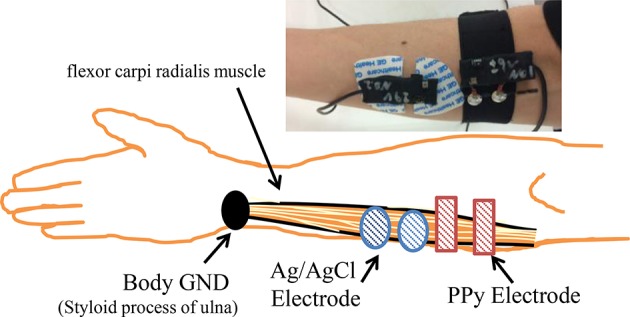
**Measurement settings of the electrodes**.

To investigate the quality of the signals measured by the proposed sensor, we carried out simultaneous measurement of sEMG with the PPy electrodes and the widely used wet electrodes, the Ag/AgCl electrodes with electrolytic gel (GE Healthcare, Japan) from the same muscle fibers. The measurement settings are shown in Figure 5. Four able-bodied subjects participated in the first experiment. Each subject was measured three times. The sampling rate was 1600 Hz and the quantization bit length was 16. The correlation coefficient of the root mean square (RMS) of the signals measured by the two kinds of electrodes and the FFT results were calculated and compared.

### Pattern recognition for myoelectric control

**Figure 6:**
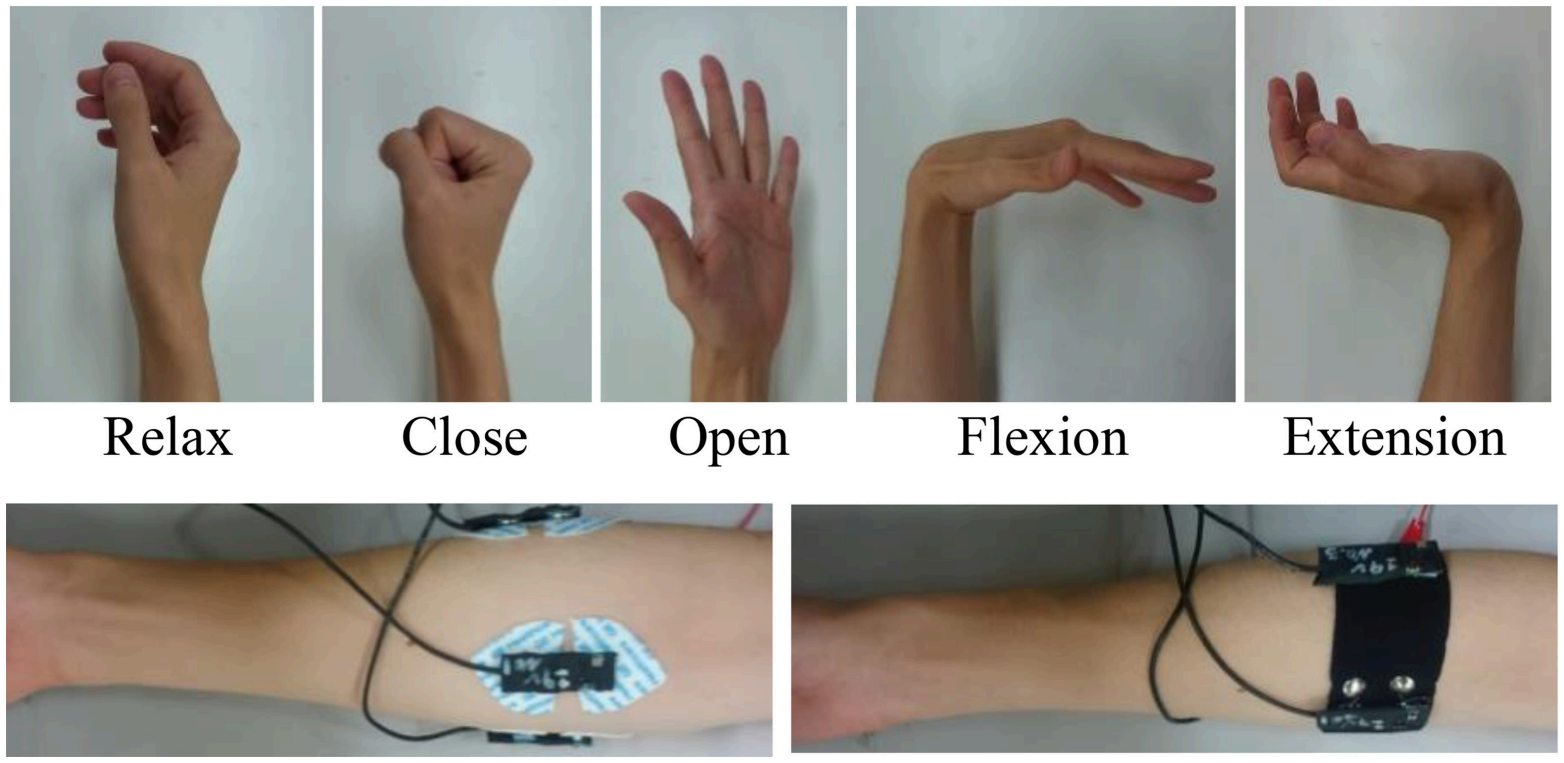
**Tasks of the experiment and position of the electrodes**.

To test the practicality of the PPy electrode, a pattern recognition experiment was conducted. In this second experiment, the sEMG signals were used for classification of multiple hand and wrist movements. Figure 6 shows the five basic motions selected for myoelectric control.

The 3-channel sEMG sensor band with the same structure as shown in Figure 3 was worn below the elbow, and the wet Ag/AgCl electrodes for comparison were adjusted to the same positions (Figure 6). The basic architecture of the pattern recognition algorithm was developed in the preceding study (Jiang et al., 2014a). The data were sampled at 1600 kHz. After A/D conversion, the SMG data were segmented into 128 ms epochs every 10 ms and FFT was performed on each epoch. The power spectrum was smoothed by the moving average. The powers of frequencies 31, 55, 78, 102, 148, 195, 258, and 320 Hz were extracted as features for input into the artificial neural network (ANN). The ANN is a 3-layer feed-forward neural network with 24 nodes in the input layer, 32 nodes in the hidden layer, and 5 nodes in the output layer.

Six able-bodied subjects participated in the experiment. The ANN was trained beforehand for each subject with a back propagation algorithm. The subjects then performed each of the five tasks for 2 s, during which the sEMG was classified 200 times. Each task was performed three times by each subject. The ratio of correct recognition to total recognition was used as the metric to compare the effectiveness of the electrodes.

### Results

**Figure 7:**
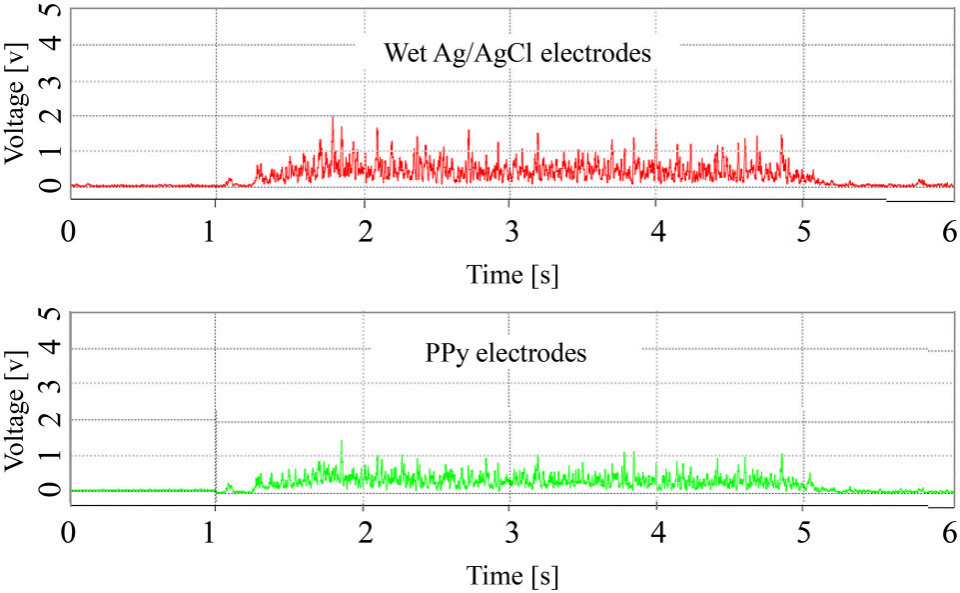
**Representative RMS of sEMG signals**.

**Figure 8:**
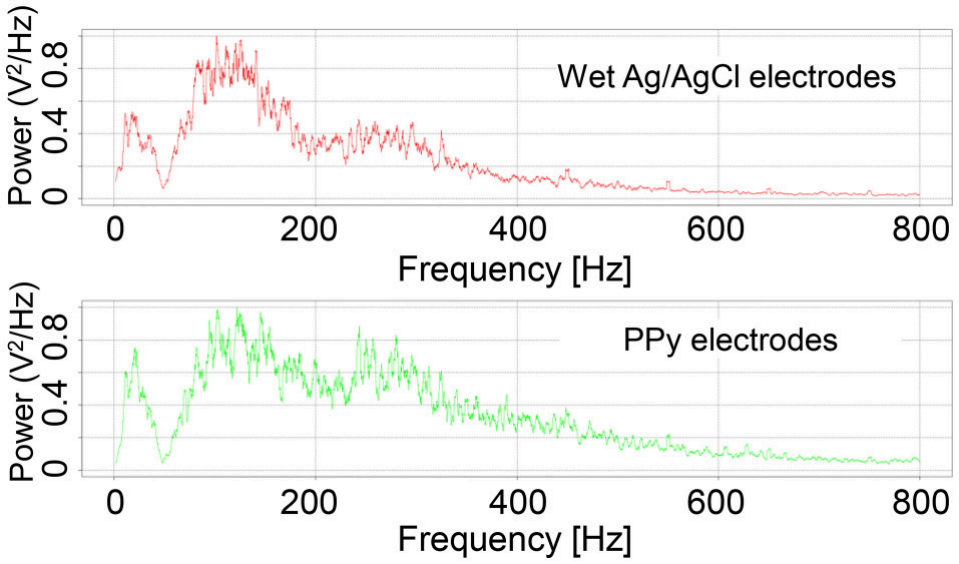
**Representative FFTs of sEMG signals**.

Examples of the RMS of the signals are shown in Figure 7. Although the amplitude of the signals measured by the wet Ag/AgCl electrodes was slightly larger, the wave shape of the signals is quite similar, with respect to the PPy electrodes. The correlation coefficients of the 12 tests are listed in Table 1. Individual differences were significant, with Subjects 1 and 2 being lower and Subjects 3 and 4 being higher, but the average correlation coefficients of the 4 subjects were all above 0.82. The overall average correlation coefficient of the 4 subjects was 0.87 (*SD* = 0.065, *n* = 12). Examples of the FFT results are shown in Figure 8. The power spectra of the signals measured with the two kinds of electrodes were very similar in the low-frequency area under 150 Hz. In the high-frequency area over 150 Hz, the power of the signals measured with the PPy electrodes was higher than that with the wet Ag/AgCl electrodes.

**Table 1:** **Correlation coefficients of the 12 tests**.

**Subject 1**			**Subject 2**			**Subject 3**			**Subject 4**		
0.82	0.83	0.87	0.76	0.87	0.83	0.83	0.85	0.88	0.97	0.96	0.96

The results of the pattern recognition are listed in Table 2. Of these tasks, Relax was recognized most accurately at nearly 100%. The other four tasks were recognized with similar accuracies around 85%. The recognition accuracies of each subject were different but consistent, with both sets of accuracies with the different electrodes being relatively lower or higher for each subject. Although the recognition accuracy was quite different according to the subject and the task, no consistent difference was observed in the accuracy with respect to the electrode types. The mean recognition accuracies for each subject showed little difference between the types. The accuracy difference was due much more to the subject difference than to the electrode types.

**Table 2:** **Results of pattern recognition**.

**Task**	**Subject A**		**Subject B**		**Subject C**		**Subject D**		**Subject E**		**Subject F**		**Mean**	
	Wet^*^	PPy^*^	**Wet**	**PPy**	**Wet**	**PPy**	**Wet**	**PPy**	**Wet**	**PPy**	**Wet**	**PPy**	**Wet**	**PPy**
Relax	100	100	99.3	100	99.5	100	99.7	99.8	100	99.2	99.8	100	99.7	99.8
Close	87.2	89.2	86.7	82.5	98.2	90.8	86.3	85.7	88.8	90.2	89.2	87.2	89.4	87.6
Open	78.3	93.5	87.7	83.5	82.8	83.2	82.1	84.5	86.2	87.9	87.5	74.3	84.1	84.5
Flexion	87.8	74.7	90.0	86.5	93.7	96.5	85.4	87.2	93.5	86.7	77.7	82.8	88.0	85.7
Extension	89.7	91.5	76.8	69.2	83.2	89.7	79.2	78.8	85.7	89.4	85.5	88.3	83.3	84.5
Mean	88.6	89.8	88.1	84.3	91.5	92.0	86.5	87.2	90.8	90.7	87.9	86.5	88.9	88.4

* *wet, wet Ag/AgCl electrodes; PPy, PPy electrodes*.

### Discussion

PPy nonwoven sheet was adopted as the material for constructing sEMG electrodes that address the problem of customization to improve the usability and practicality of myoelectric control. The size and shape of the sheet are easily adjusted to amputees whose stumps have different residual muscles. The PPy electrodes were compared with the widely used wet Ag/AgCl electrodes to investigate the signal quality and to confirm their feasibility for myoelectric control of multi-DoF prosthetic hands. The simultaneous measurements with the two types of electrodes suggest that signal quality was quite similar and the difference for myoelectric control was negligible. The displacement of electrodes and electrode material were two considerable reasons for the difference in the correlation coefficient. The displacements in the longitudinal direction with respect to the muscle fibers may affect the EMG characteristics (Young et al., 2012). It was hard to place the electrodes onto the skin strictly along the muscle fiber. Interface capacitance existed between skin and the dry PPy electrodes without utilize an electrolyte, as in the gel of the wet Ag/AgCl electrodes. The different electrical property of the electrode materials might contribute to the difference in the sEMG signals.

From the results of the general FFT and the conventional ANN with a simple structure implementable on a microprocessor, the average recognition accuracy of the PPy electrodes for 5 classes of movement was nearly 90%, which was comparable to the average accuracy of the wet Ag/AgCl electrodes. These experimental results show that the proposed PPy electrodes, which are highly usable and customizable, are as effective as the wet electrodes for myoelectric control. Besides the conditions of the subject, the performance of EMG based motion classification mainly depends on measurement, feature extraction, and classification. Many methods have been reported for the analysis and classification of sEMG signals (Chowdhury et al., 2013). The methods, with their advantages and disadvantages, lead to different classification results which might further elucidate the characteristics of the measurement method. We adopted the general FFT and the conventional ANN in this study to show the performance of the PPy electrodes for the practical control of prosthetic hand. Future work will include other methods to further investigate the characteristics of the proposed PPy electrodes.

## Relation between sEMG quality and source impedance

The electrodes act as a transducer by converting ionic current within the tissue to electrical current to the amplifier. The source impedance, including the tissue impedance, the skin-electrode impedance, and the electrode impedance, is one of the most important factors affecting the S/N ratio. The mismatch of source impedance decreases the common mode rejection ratio (CMRR), which degrades the S/N ratio of the signal (Degen and Jackel, 2008). The proposed PPy electrodes can be easily cut into different shape and size with different conductivity and skin-electrode impedance. The balance of source impedances may be a criterion in preparation of the PPy electrodes. An experiment was therefore conducted to investigate the relation between sEMG quality and source impedance.

### Analysis of signal correlation

**Figure 9:**
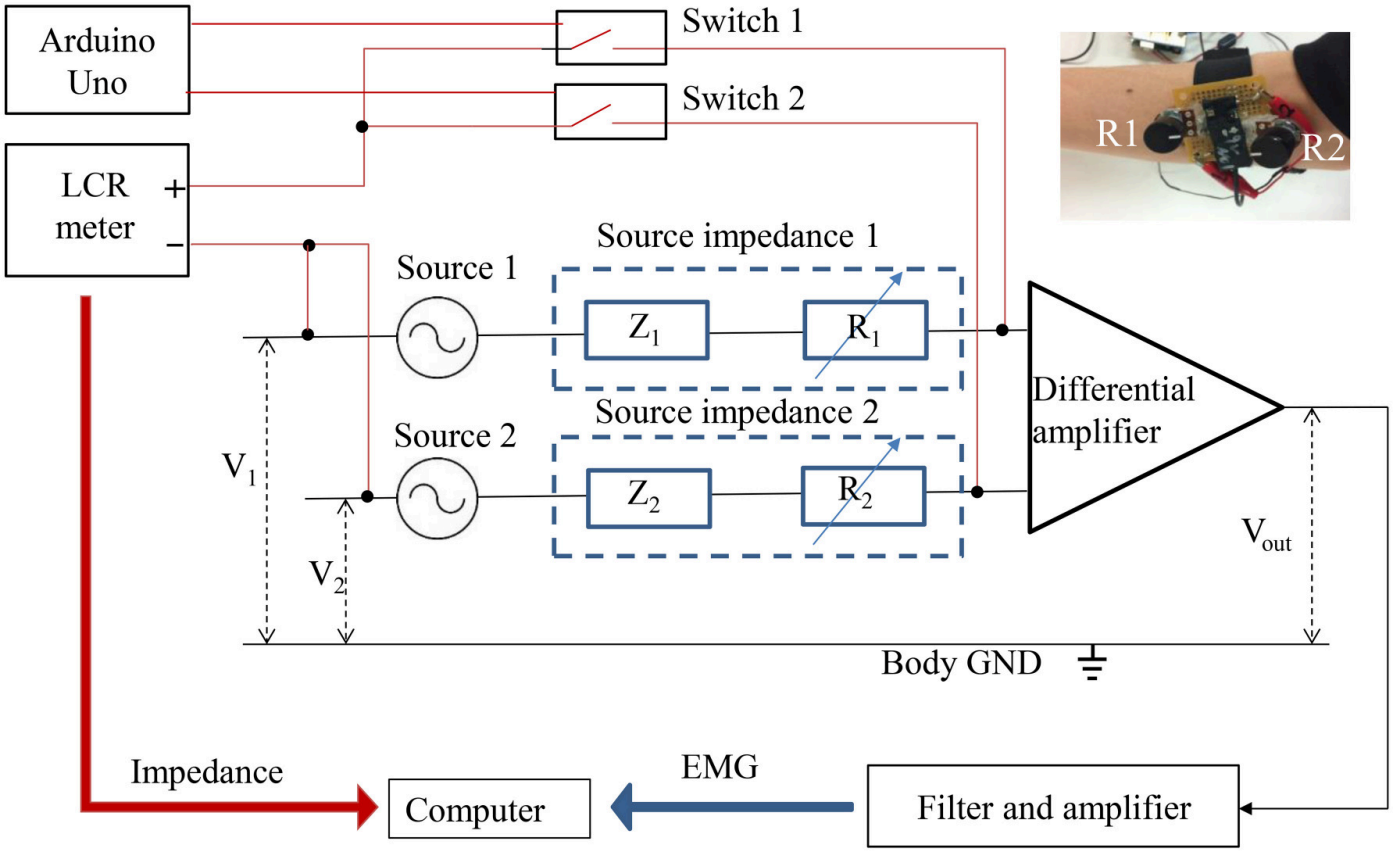
**Simultaneous measurements of EMG and impedance**. The red line indicates the impedance measurement, and the black line indicates the EMG measurement.

The experimental system for simultaneous measurement of the sEMG and the source impedance is shown in Figure 9. Sources 1 and 2 are the origins of EMG signals. Z_1_ and Z_2_ indicate the combination of tissue impedance, skin-electrode impedance, and electrode impedance. R_1_ and R_2_ are variable resistances (0–200 kΩ) to enable manual adjustment of the source impedances, since the source impedances to the differential amplifier include variable resistances. By manually adjusting R_1_ and R_2_ to increase the imbalance of the source impedances, the CMRR declines, thus causing a decrease in the S/N ratio. The photo at the top right of Figure 9 shows the variable resistances installed before the amplification circuit. An LCR meter (ZM 2371, NF Corporation, Japan) was used to measure the magnitude and phase angle of the source impedances at 300 Hz and 1 Vrms. Switches 1 and 2, controlled by Arduino Uno (Arduino, Italy), switch the measurement of source impedance 1 and source impedance 2 with the LCR meter. It is important to note that the source impedance exhibits a number of amplitude- and frequency-dependent nonlinear effects (Dorgan and Reilly, 1999). The source impedances at 300 Hz and 1 Vrms were measured as representative values, since measurements at multiple frequencies would cost time and decrease the simultaneity between impedances and the sEMG. During the impedance measurement, the 1 Vrms input signal was also amplified, which caused amplifier saturation. The saturation started with the start and ended with the end of the input signal. The output of the differential amplifier during the impedance measurement was therefore excluded from the sEMG analysis.

**Figure 10:**
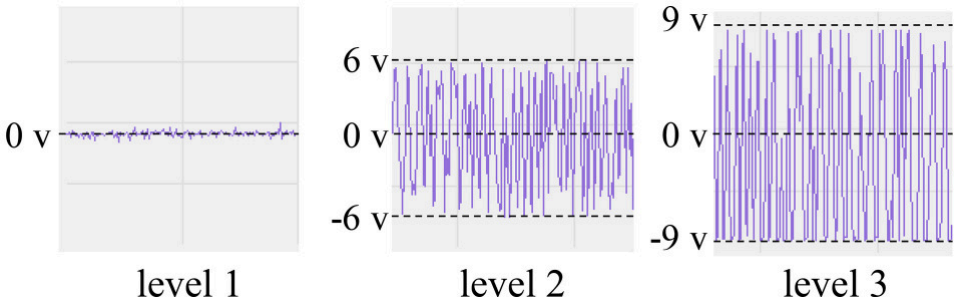
**Noise levels during the rest state**.

The sEMG of an able-bodied subject doing flexion was measured by placing the PPy electrodes above the digitorum profundus muscle. To investigate the relation between the source impedance balance and the S/N ratio, we adjusted the noise level by tuning R_1_ and R_2_ during the rest state, and then measured the sEMG and the source impedances at the noise level. The three noise levels are illustrated in Figure 10. Levels 1, 2, and 3 show little noise, noise in the range of (−6 v, 6 v), and noise in the range of (−9 v, 9 v), respectively. At each level, the sEMG and source impedances were measured ten times. The differences of the magnitude and phase angle were used as an index of source impedance imbalance, and the FFT of the sEMG signals were calculated to analyze the noise.

### Results

Table 3 shows the source impedance imbalance at the three noise levels. Both the magnitude differences and the phase angle differences are significant. At noise level 1, the imbalance of magnitude is around 1500 Ω and the phase angles are nearly the same. To increase the noise to levels 2 and 3, the imbalance was increased in magnitude to around 8000 and 20,000 Ω, and the phase angle was increased to around 10 deg and 30 deg, respectively. Accordingly, the SD of the imbalances in the magnitude and phase angle also increased with the increase of noise level.

**Table 3:** **Magnitude and phase angel differences at the three noise levels (Impedances were measured at 300 Hz and 1 Vrms)**.

	**Noise level 1**		**Noise level 2**		**Noise level 3**	
	**Magnitude (Ω)**	**Phase angle (degree)**	**Magnitude (Ω)**	**Phase angle (degree)**	**Magnitude (Ω)**	**Phase angle (degree)**
1	1300	0.6	5300	6.4	18,300	21.5
2	3000	0.2	6600	7.4	21,700	22.9
3	3700	0.7	7500	5.5	12,700	20.4
4	200	3.0	7200	4.8	16,500	22.4
5	1400	4.1	7500	5.5	11,800	40.3
6	600	0.4	9400	12.0	19,000	40.8
7	1100	1.0	8300	11.6	28,800	39.9
8	1600	0.5	9500	12.0	29,600	41.1
9	1200	0.3	8700	12.2	24,300	41.8
10	1500	0.6	8800	11.8	29,800	40.5
Mean	1560	1.2	7880	8.9	21,250	33.2
SD	1047	1.3	1320	3.2	6734	9.8

**Figure 11:**
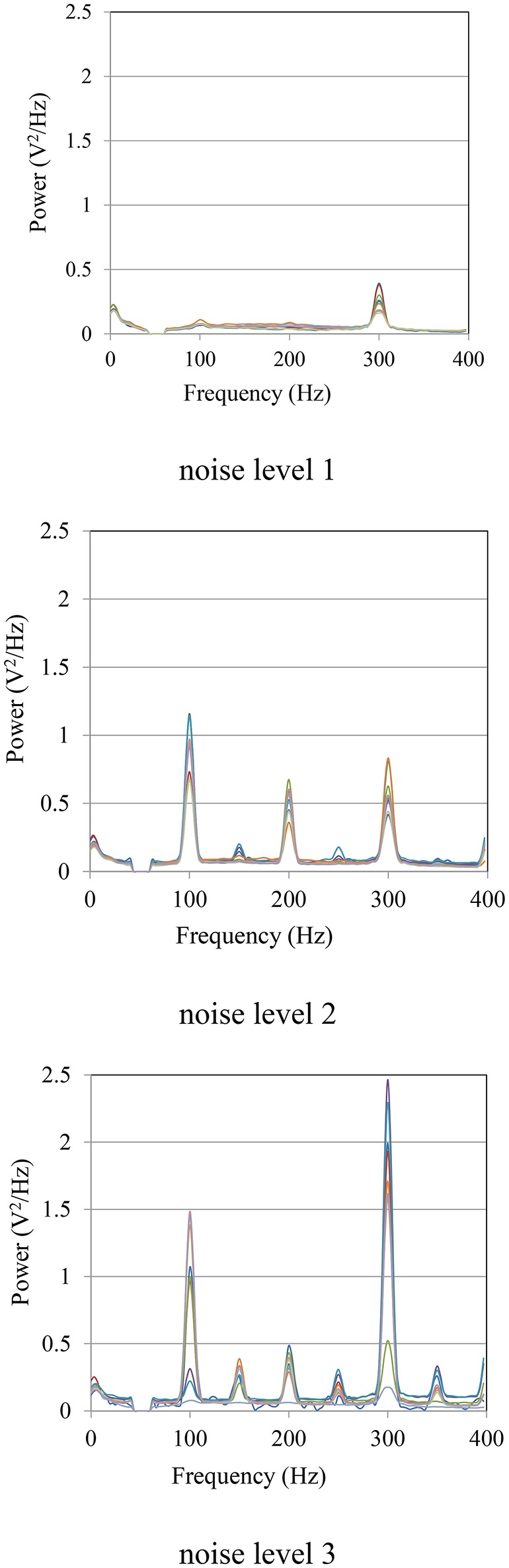
**Power spectra of sEMG signals at noise levels 1, 2, and 3**.

The power spectra of the measured sEMG signals at different noise levels are shown in Figure 11. The noise mainly came from the power line radiation (50 Hz) and its harmonics, and the measurement frequency (300 Hz) of the LCR meter. At noise level 1, the noise was mainly caused by the LCR meter at 300 Hz. With the decline of the CMRR due to the imbalance of source impedances, more noise was mixed in with the sEMG signals. Especially at the frequencies of 100, 200, and 300 Hz, the power of the noise was much higher than that of the sEMG signal.

### Discussion

An important factor of the dry electrodes is their ability to maintain electrical contact with skin, since the electrical contact affects the electrode-skin impedance and the source impedance balance. The dry electrodes do not utilize an electrolyte, as in the gel of the wet Ag/AgCl electrodes. High interface capacitance therefore exists between the skin and the electrode, so that the source impedances are larger when using dry electrodes than those when using wet electrodes. A comparison study (Searle and Kirkup, 2000) investigated the contact impedances, the resistance to electrical interference, and the movement artifacts of the dry and wet electrodes. However, the relation between contact impedances and S/N ratio was not quantitatively measured.

In this study, the simultaneous measurement of the source impedance balance and the sEMG by using the switching circuit suggest that the imbalances in the magnitude and phase angle of the source impedances were the reason for the increase of noise level. By adjusting the variable resistances to unbalance the source impedances, the CMRR of the differential amplifier declines, and thus the common mode noises, caused by harmonics of the power line radiation and the measurement signal, were also amplified. Conversely, if the source impedances are balanced, the S/N ratio can be improved. Source impedances, rather than the shape and the size of the electrodes, are important for sEMG measurement. Therefore, two electrodes connected to the same amplifier do not have to have the same shape and size as long as the source impedances are close. The proposed flexible PPy electrodes provide high freedom of shape and size adjustment to fit a specific amputee.

## General discussion

We developed a novel PPy electrode for the convenient measurement of sEMG signals. The conductive nonwoven fabric sheet is easily cut and sewn to fit an individual. Currently, most amputees need to be checked as to whether they are suitable to use a myoelectric prosthetic hand at hospitals or other medical institutions. Before amputees get myoelectric prosthetic hands, which are custom-made according to the prescription by the prosthesis therapist, they are required to participate in training up to 2 months in order to generate a stable sEMG signal. This process decreases the accessibility of myoelectric prosthetic hands, especially for those who are poor and living in undeveloped areas. The goals of our current study and our preliminary studies (Jiang et al., 2014a, 2015) are to develop a low-cost and highly usable myoelectric prosthetic hand system in order to increase its accessibility, both economically and technically. In order to provide the technology and the opportunities necessary for people with disabilities to maximize their quality of life by using prosthetic hands, the whole system including the sEMG measurement should be conveniently adaptable to any person, since each person represents different conditions. Usability is one of the main factors that affects prosthesis acceptance or rejection (Biddiss and Chau, 2007; Vasluian et al., 2013). The trial use experiment illustrated that the PPy electrodes provide a convenient and customizable measurement method for amputees. The comparison with wet Ag/AgCl electrodes suggest that the PPy electrodes are usable and capable of recording sEMG signals for myoelectric control.

To address this challenge of sEMG measurement, many kinds of electrode construction using pure or mixed materials such as stainless steel, Si3N4, Ag/AgCl, and Au have been proposed (Searle and Kirkup, 2000). Recently, conductive polymers, especially PEDOT-PSS, have been widely studied as promising bioelectrode materials (Richardson-Burns et al., 2007; Lang et al., 2009; Tsukada et al., 2012; Isik et al., 2015; Nagamine et al., 2016; Pani et al., 2016; Papaiordanidou et al., 2016). PEDOT-PSS has highly electroconductive and biocompatible characteristics that meet the requirements for biomedical electrode materials (Takamatsu et al., 2015; Pani et al., 2016). However, because it is more water-soluble and hygroscopic, PEDOT-PSS exfoliates from the substrates and absorbs water in wet conditions, which will reduce its mechanical strength and electrical characteristics (Elschner et al., 2010). Although the conductivity is lower, polypyrrole is more stable than PEDOT-PSS in the sEMG measurement environment, in which the electrode may become wet with sweat. The low conductivity can be compensated to some extent by the use of active electrodes (where buffering and amplification are performed at the electrode site) and high-quality amplifiers. The experimental comparison with wet Ag/AgCl electrodes suggests that although the conductivity of the PPy electrodes is much less than that of the wet Ag/AgCl electrodes, the signal quality was quite similar and negligible for myoelectric control.

One major cause of the noise in the sEMG is the common mode interference that is mixed in the signal when the CMRR declines due to the skin-electrode impedance change (Yokus and Jur, 2016). The switched simultaneous measurements of the sEMG and source impedances suggest the importance of source impedance balance for signal quality. Theoretically, any conductive material has the potential to become an electrode if the amplifier is ideal, with infinitely great input impedance. Since such amplifiers do not exist, most of the studies focus on the electrical property in order to develop electrodes with skin-electrode impedance that is as low as possible (Hahne et al., 2016). However, for a practical use such as myoelectric control, a tradeoff exists between reducing the contact impedance and improving the usability and customizability. The electromechanical stability of the contact between sEMG electrodes and skin directly affects the quality of the signal (Roy et al., 2007), but the stability is hard to guarantee when using metallic electrodes to control a prosthetic hand in a real-life environment, since impedance changes can be induced by motion (Cömert and Hyttinen, 2014). With the improvement in electronic devices, more flexible conductive materials with relatively low conductivity can be alternatives to developing practical surface bioelectric electrodes. The simultaneous measurements of the source impedance balance and the sEMG also show the possibility of improving the S/N ratio by balancing the source impedances. If the source impedances can be monitored and adjusted with variable resistances to decrease the impedance differences, the CMRR of the differential amplifier could be improved to exclude the common mode interferences.

In preparation of the PPy electrodes, source impedance and interelectrode distance (Young et al., 2012) are two important factors to determine their shape and size. Desirable characteristics of electrode-skin impedance are a low value of the impedance and a balance of values between the electrodes. Small electrodes with less contact area may lead to high electrode-skin impedance and the difference of electrode-skin impedance may cause source impedance unbalance. High density electrodes are often adopted in scientific studies to obtain fine EMG characteristics or to improve the robustness by redundancy. However, in practical control of prosthetic hands, most of which have <5 DoFs currently (Belter et al., 2013), three effective channels of sEMG signal are enough. Another non-negligible factor is the conditions of the amputees (i.e., the number and distribution of residual muscles). The flexible PPy electrodes can be easily cut into the shape and size that fit a specific amputee. Metal electrodes also can be customized into different shapes and sizes. But metallic processing is not as easy as cutting a fabric sheet. And metal electrodes are not flexible to fit closely to the body.

In sum, the PPy electrodes investigated here showed their usability and effectiveness in sEMG recording for myoelectric control of prosthetic hands. Nevertheless, because the trial use by amputees and the experiments were carried out over a short period of time (<1 day for the trial use and 1 week for the experiments), the durability needs to be further validated. The stability of conductive polymers including PPy and PEDOT:PSS is different with respect to dopants, synthesis methods, environment, and usage in applications (Yamato et al., 1995; Cui and Martin, 2003; Jang et al., 2004; Khodagholy et al., 2011; Andreoli et al., 2013). To the best of our knowledge, there is no studies comparing PPy and PEDOT:PSS in the form of fabric sheet. Since PPy is stable under wet and humid conditions, the durability is expected to be better than that of electrodes made of PEDOT:PSS. Furthermore, the properties of the nonwoven fabric sheet, including the surface texture and the geometric configuration, need to be further investigated. The performance of the PPy electrodes may be improved by optimizing their properties.

## Conclusion and outlook

We have proposed a sEMG sensor using PPy electrodes for practical myoelectric control of prosthetic hands in daily life. The flexible electrodes are highly customizable to fit the size and shape of different stumps. The PPy electrodes enable highly usable and effective sEMG measurements in real-life settings. By sewing the electrodes on elastic webbing, it is easy to construct a band that can be put on one-handed to record multiple channel sEMG signals. Experiments comparing PPy electrodes with wet Ag/AgCl electrodes suggest that the PPy electrodes are as effective as the widely used wet Ag/AgCl electrodes for myoelectric control of prosthetic hands. The simultaneous measurements of source impedances and sEMG suggest that the source impedance imbalance is a main reason for the low S/N ratio, and so the S/N ratio may be improved by balancing the source impedances. We will continue to investigate the durability of the sEMG sensor using PPy electrodes and to improve it by optimizing the properties of the fabric sheet. Long-term home trial use together with prosthetic hands by amputees is under planning. PPy electrodes with different surface textures and geometric configurations will also be tested in the near future.

## Author contributions

YJ, MT, BL, and HY conceived the study concept. YJ, MT, and HY developed the electrode with the polypyrrole-coated nonwoven fabric sheet. YJ and MT designed and executed the experiments. YJ, MT, BL, and HY interpreted the experimental data. YJ drafted the manuscript. MT, BL, and HY revised the manuscript.

### Conflict of interest statement

The handling Editor declared a shared affiliation, though no other collaboration, with one of the author BL and states that the process nevertheless met the standards of a fair and objective review. The other authors declare that the research was conducted in the absence of any commercial or financial relationships that could be construed as a potential conflict of interest.
